# Experimental crossover study on the effects of withholding feed for 24 h on the equine faecal bacterial microbiota in healthy mares

**DOI:** 10.1186/s12917-020-02706-8

**Published:** 2021-01-05

**Authors:** Jaclyn A. Willette, Dipti Pitta, Nagaraju Indugu, Bonnie Vecchiarelli, Meagan L. Hennessy, Tamara Dobbie, Louise L. Southwood

**Affiliations:** 1 Departments of Clinical Studies, New Bolton Center, University of Pennsylvania School of Veterinary Medicine, 382 West Street Rd, Kennett Square, PA 19348 USA; 2 Present address: Department of Clinical Sciences, Michigan State University, East Lansing, MI USA

**Keywords:** Microbiota, Gastrointestinal, Equine, Colic

## Abstract

**Background:**

An association between equine gastrointestinal disease causing colic signs and changes in faecal bacterial microbiota has been identified. The reasons for these changes and their clinical relevance has not been investigated. Withholding feed, which is an integral part of managing horses with colic, may contribute to the observed changes in the microbiota and impact interpretation of findings in horses with colic. Study objectives were, therefore, to determine the effect of withholding feed for 24 h on equine faecal bacterial microbiota in healthy mares to differentiate the effects of withholding feed from the changes potentially associated with the disease.

**Results:**

Species richness and Shannon diversity (alpha diversity) were significantly lower at the late withheld (10–24 h post withholding feed) and early refed (2–12 h post re-feeding) time points compared to samples from fed horses (*P* < 0.01). Restoration of species richness and diversity began to occur at the late refed (18–24 h post re-feeding) time points. Horses having feed withheld had a distinct bacterial population compared to fed horses (beta diversity). Bacteroidetes *BS11* and Firmicutes *Christensenellaceae*, *Christensenella*, and *Dehalobacteriaceae* were significantly increased in horses withheld from feed primarily during the late withheld and early refed time points. Bacteroidetes *Marinilabiaceae* and *Prevotellaceae*, Firmicutes *Veillonellaceae*, *Anaerovibrio*, and *Bulleidia*, and Proteobacteria *GMD14H09* were significantly decreased in horses with feed withheld at late withheld, early refed, and late refed time periods (*P* < 0.01). Changes in commensal gut microbiota were not significant between groups.

**Conclusions:**

Withholding feed has a significant effect on faecal bacterial microbiota diversity and composition particularly following at least 10 h of withholding feed and should be taken into consideration when interpreting data on the equine faecal bacterial microbiota in horses.

## Background

The association between gastrointestinal disease and the faecal bacterial microbiota is an area of active research across species. Humans and cattle with chronic gastrointestinal disease have a decrease in bacterial species richness and diversity compared to healthy subjects [1–5]. Recently published data revealed that horses presenting to a tertiary referral hospital for colic had significantly decreased admission faecal or colonic bacterial species richness and diversity and a distinct bacterial population compared to horses presenting for an elective surgical procedure [6, 7]. In another related study, horses with chronic colic (> 60 h duration) had significantly decreased faecal bacterial diversity compared to horses with acute colic (< 60 h) [8]. It is plausible that alterations in the faecal microbiota may be associated with intestinal dysbiosis and inflammation leading to signs of colic [9]. Some of the differences in the microbiota observed in horses admitted for colic (compared to an elective surgical procedure) were consistent with gastrointestinal disease in horses and other species including humans [10–14]. Horses presenting for colic appear to have a microbiota that is distinct from horses without apparent gastrointestinal disease. These findings could potentially contribute to our understanding of the causes of colic and lead to prevention strategies and treatment options. However, there are numerous possible reasons for the results of the previously noted studies that were not assessed. Variables include the fact that horses showing colic signs likely have had feed withheld prior to admission to referral hospitals, whereas horses admitted for an elective surgical procedure may not have had feed withheld. Similarly, horses with chronic colic likely have feed withheld for longer than horses with acute colic. Withholding feed may affect the faecal microbiota and its effect needs to be determined in healthy horses to better interpret studies on the microbiota of horses with colic.

To the authors’ knowledge, there is little information on the effect of withholding feed on the equine faecal microbiota. Studies have shown that the there is a decrease in diversity of the equine faecal microbiota associated with domestication and captivity [15] and that the faecal microbiota varies with season, ambient weather conditions, and feed [16]. A study by Schoster et al. investigating the effects of transport, fasting, and anaesthesia on the faecal microbiota of healthy adult horses found that horses that were fasted for a relatively short period of time (12 h) post-transportation had an alteration in the relative abundance of bacterial taxa comprising the microbiota (decrease in Firmicutes and increase in Proteobacteria) with no change in diversity and species richness [17]. The duration of fasting in this study was relatively short and confounded by the effects of transportation. The duration of time between transportation and fasting differed for each horse adding to the study variability and there was no control population of horses. Understanding the faecal microbiota changes associated with management, including withholding feed, is necessary to interpret findings in horses with colic. Our null hypothesis was that withholding feed would have no significant effect on faecal bacterial microbiota richness, diversity or composition in healthy horses. The specific aims designed to test our hypothesis were to compare the richness, diversity, and relative abundance of bacterial taxa comprising the faecal bacterial microbiota in healthy mares withheld from feed or fed ad libitum.

## Results

### Sequencing information

A total of 23,212,335 million raw reads were generated from a total of 392 samples (8 samples could not be collected because of a lack of faeces in the rectum, Supplemental Table S1), with mean (± SD) of an average of 59,215 (± 15,176) reads per sample. Less than 100 reads per sample were observed in the blank samples (two DNA blanks and three PCR blanks) and they were dropped from the analysis. This produced a total of 67,700 ASV. Representative sequences from the ASV were assigned to 24 bacterial phyla. A total of 318 genera were observed in this study.

### Community comparison

**Fig. 1:**
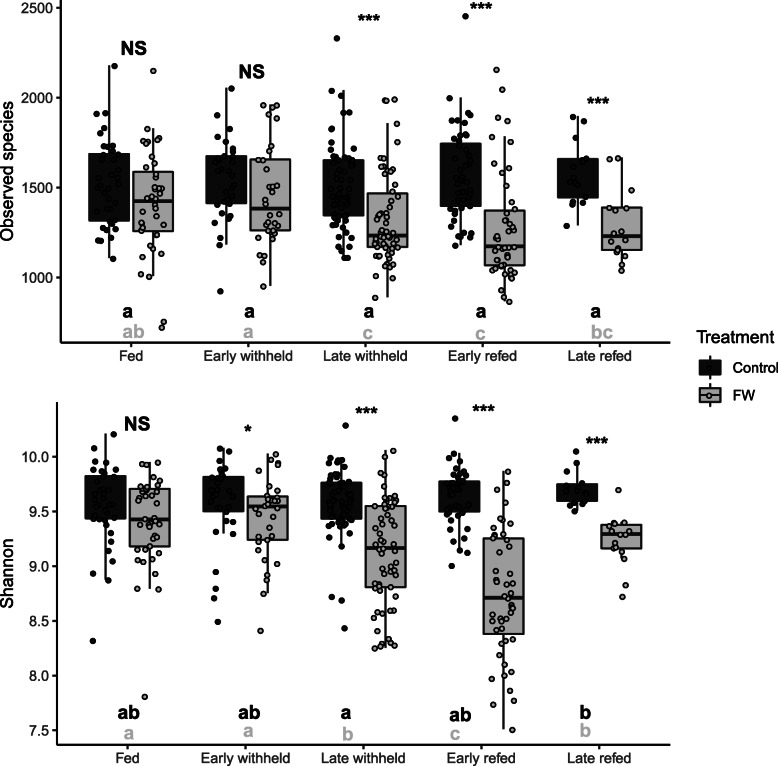
Boxplots showing species richness (observed species) and diversity (Shannon) comparing horses in the control (fed, dark grey) and feed withheld (FW, light grey) groups during the fed, early withheld, late withheld, early refed, and late refed time periods (see Fig. 6 for definitions). The species richness and diversity were significantly decreased in the FW group during the late withheld, early refed, and late refed time periods. NS, not significantly different; * and ***, significant difference between control and FW groups (*P* < 0.05 and *P* < 0.001, respectively). Different letters represent significant differences between time periods within the same group (*P* < 0.05)

**Fig. 2:**
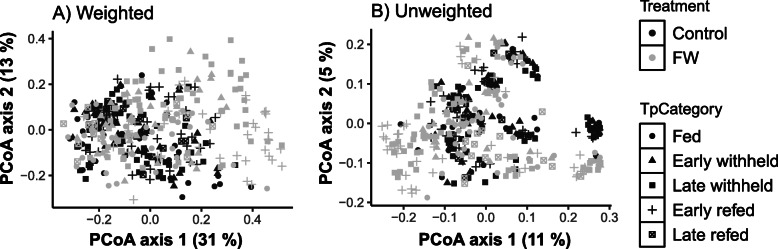
Measurement of bacterial community composition (beta diversity) of the faecal bacterial microbiota for the effect of treatment (fed control horses [black shapes] versus feed withheld, FW [grey shapes]) and time period category (see Fig. 6 for definitions). The principle coordinate analysis (PCoA) plot shows weighted (**a**, relative proportions of commonly present 16S rRNA bacterial sequences) and unweighted (**b**, presence-absence information of 16S rRNA bacterial sequences) UniFrac distances between samples. Samples that are more similar are located more closely to one another, and dissimilar samples are further apart. Note that the grey solid squares (FW horses during the late withheld time period) and grey plus-signs (FW horses during the early refed time period) are spread out away from the cluster with the black shapes (fed control horses during all time periods) and grey circles, triangles, and non-sold square (FW horses during the fed, early withheld, and late refed time periods, respectively). Pairwise comparisons of weighted data showed that there was no significant difference between groups during the fed time period. Significant differences were observed between groups during the early withheld (*P* = 0.021) and the late withheld, early refed, and late refed time period (*P* = 0.001)

**Fig. 3:**
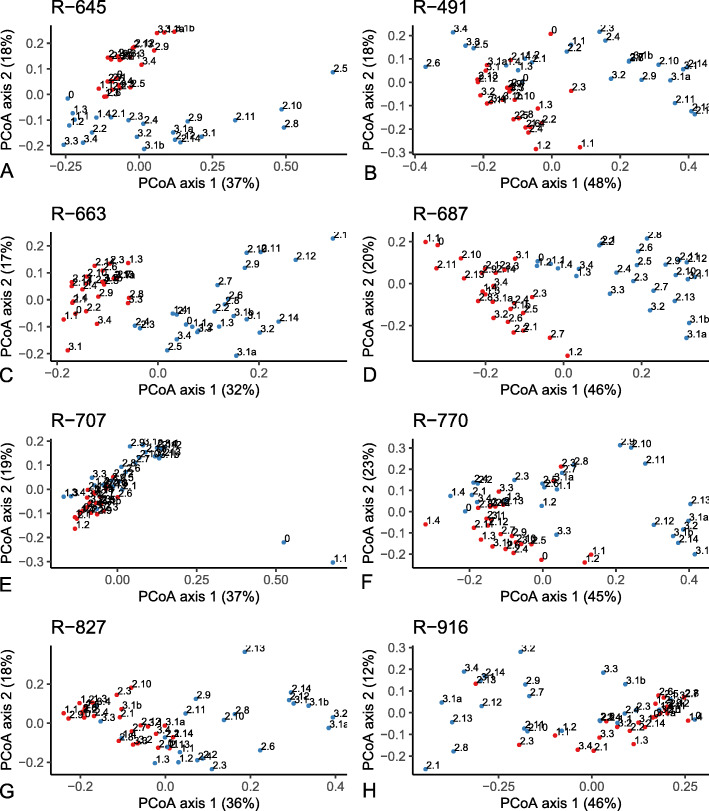
PCoA for each horses illustrating the individual horse effect of withholding feed on the beta diversity of the two groups (control red; feed withheld blue). Numbers associated with the data points represent the samples shown in Fig. 6. Of note is that in general the control samples (red) are more tightly clustered and the data points move further away from control cluster with increase in duration of feed withholding time i.e. the points 2.10, 2.11, 2.12 [late withheld], 2.13, 2.14, 3.1, 3.1a, 3.1b [early refed] are generally furthest from the control samples and represent the late withholding-early refeeding period (20–30 h post beginning withholding feed). Data point 3.4 (study completion) is closer to the control cluster suggesting that within 24 h the microbiota returns to the control sample population. Also, of note is the 0 and 1.1 time points for the control group which were taken when the horse was first brought into the stall; the effect of moving horses from outside to inside warrants further analysis. All horses had a similar PCoA pattern

The alpha diversity as measured by species richness and Shannon diversity (Fig. 1) showed significant (*P* < 0.05) differences between horses that were withheld from feed compared to those same horses when they had ad libitum access to feed. There were no differences between the two groups at time 0 and during the fed time period. Compared to fed (control) horses, species richness and diversity were significantly decreased in horses when feed was withheld at the late withheld, early refed, and late refed periods. Of note is that while observed species and Shannon diversity values remained unchanged for fed (control) horses during the entire study period, decreases were noted for these indices when horses were withheld from feed, for late withheld, early refed, and, for observed species, late refed periods. Based on the weighted (commonly present bacterial populations) and unweighted (presence and absence information of bacteria) UniFrac analysis, there was a significant difference (*P* < 0.001; PERMANOVA test) in the bacterial community composition between the horses when they were fed (control) compared to when the same horses were withheld from feed (Figs. 2 and 3a-h, Supplemental Information Fig. S1). There was a significant interaction between group (control v. feed withheld) and time period (*P* < 0.001). Pairwise comparisons of weighted data showed that there was no significant difference between groups during the fed time period. Significant differences were observed between groups during the early withheld (*P* = 0.021) and the late withheld, early refed, and late refed time period (*P* = 0.001).

Of note is that there was no significant effect on the faecal microbiota of bringing the horses into a stall from a pasture (time point 0, *P* = 0.424) (Supplemental Information Fig. S2). The horses were maintained on free choice timothy hay regardless of housing (inside stall v. outside pasture) due to the lack of pasture in winter and early spring. There was also no significant effect of diurnal variation (day v. night) on the faecal microbiota (*P* = 0.165).

### Taxonomic distribution of bacterial communities

**Fig. 4:**
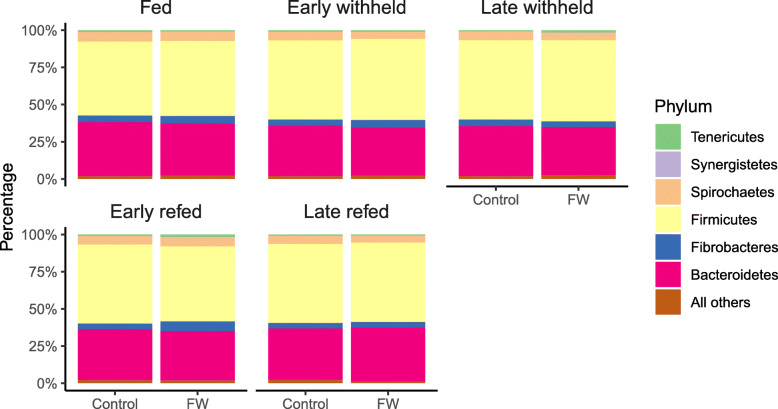
Relative abundance of each of the major phyla in the fed control and feed withheld (FW) groups at the fed, early withheld, late withheld, early refed, and late refed time periods. See Fig. 6 for definitions

Faecal bacterial communities were characterized at the level of bacterial phyla for all horses sampled across all time points. The majority of the 16S rRNA-encoding gene sequences were dominated by Firmicutes (52.4%) and Bacteroidetes (34.1%), which together comprised about 86% of the total bacterial abundance. Other phyla present in lower proportions included Spirochaetes (5.6%) and Fibrobacteres (4.6%), with Proteobacteria, Actinobacteria, and Tenericutes each individually contributing to less than 1% (Fig. 4, Supplementary Table S2).

**Fig. 5:**
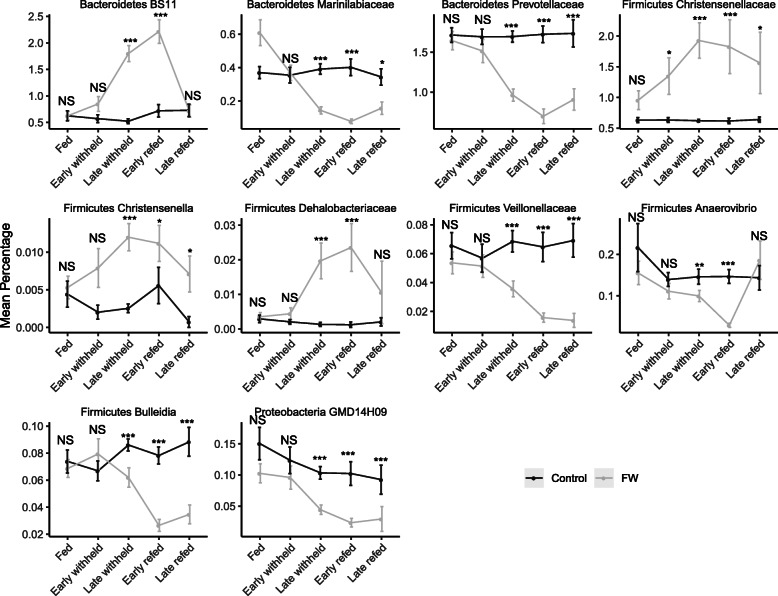
Significantly differentially abundant bacterial genera identified by ANCOM comparing fed control horses (black lines) and horses from which feed was withheld (FW, grey line) at the different time periods (see Fig. 6 for definitions). Level of significance *P* < 0.05. NS, not significantly different. *, *P* < 0.05, **, *P* < 0.01, ***P < 0.001

The mean values of relative abundance of genera that were detected at various time points (fed, early withheld, late withheld, early refed, and late refed) in both horses that received full access to feed and horses that were withheld from feed are presented (Supplementary Table S3). Using the ANCOM test, we found that ten genera were significantly different (*P* < 0.05) between horses that were withheld from feed compared to horses that had ad libitum access to feed (Fig. 5). Notably, none of these genera showed differences at fed and early feed withheld periods but showed differences at later time points (Wilcoxon test). Of these ten genera, *BS11* from Bacteroidetes and unclassified *Christensenellaceae*, *Christensenella*, and unclassified *Dehalobacteriaceae* from Firmicutes increased in horses that were withheld from feed whereas these genera remained unaltered in horses that had ad libitum access to feed. In contrast, unclassified *Marinilabiaceae* and unclassified *Prevotellaceae* from Bacteroidetes, unclassified *Veillonellaceae, Anaerovibrio*, and *Bulleidia* from Firmicutes, and *GMD14H09* from Proteobacteria showed a steep decrease from late withheld and later time points in horses that were withheld from feed whereas these genera were unaltered in horses that had unrestricted access to feed.

## Discussion

It has been proposed that gastrointestinal disease may result from disruption of complex interactions between the host and the commensal gut microbiota and withholding feed may also play an important role in these disturbances particularly in horses with gastrointestinal disease causing colic. The purpose of this study was, therefore, to investigate the changes in the faecal bacterial microbiota of healthy horses that were withheld from feed for 24 h compared to horses that were fed. The results of this study revealed that the bacterial community composition in horses withheld from feed was substantially different to that of horses who were maintained on their normal diet. Horses withheld from feed had a decrease in bacterial composition and overall species numbers. Findings of this research indicate that horses withheld from feed demonstrate significant changes in their faecal microbiota, particularly after 10 h of withholding feed. The microbiota did gradually return to pre-withholding feed composition 18–24 h after re-introduction of feed.

The gastrointestinal microbial communities colonizing the caecum and colon play important roles in feed digestion, offer protection from pathogen invasion, maintain the integrity of intestinal epithelium, and promote immune responses [17, 18]. While efforts to understand the composition of microbial communities colonizing the equine gastrointestinal tract have provided insights on the presence of core microbiota in horses, the functional relevance of the individual populations and their sensitivity to external perturbations such as changes in diet, management, incidence of diseases, and therapeutic interventions are not very well understood. Fasting or withholding feed is a common confounding factor and along with other potentially confounding factors and is likely to influence the gut microbiota. The effect of withholding feed on changes in the equine microbiota in isolation has not been previously explored. Schoster et al. [17] investigated the effect of fasting for 12 h on changes in microbiota along with several other factors such as transportation and anaesthesia. These authors reported no changes at the community level but reported a reduction in *Clostridiales* with fasting. It is possible that fasting for 12 h may not be sufficient to induce changes in the faecal bacterial microbiota or the effect might not be observed until after 12–24 h. Therefore, one time sampling at 12 h may not provide accurate information on changes in microbiota. It has been reported that gut microbes contribute to adaptation and survival of their host during a starved state as demonstrated in higher mortality rates in germ-free mice compared to conventional mice [19]. Further, it has been speculated that certain populations can thrive and contribute to energy conservation via alternative pathways such as nitrogen recycling, lipid metabolism, and other adaptation mechanisms to enable the host to survive in feed deprived conditions [20]. McCabe et al. [21] demonstrated that partial restriction of feed in beef cattle for 125 days reduced propionate-producing bacteria and increased fiber-digesting bacteria and methanogens, and these changes were restored to normal upon providing full-access to feed. In the current study, feed was withheld for only 24 h. Changes in the faecal bacterial microbiota were observed primarily during the late withheld (10–24 h post withholding feed) and early refed (2–12 h post refeeding) time periods with diversity and some phyla returning to fed levels by the late refed (12–24 h post refeeding) time period. While application of this information directly to equine colic may still be premature, these findings suggest that for colic patients with signs shorter than 10 h, changes in bacterial richness, diversity and composition may not simply be attributed to withholding feed or inappetence.

At the community level, our previous findings showed a lower bacterial richness and diversity in horses presenting for colic compared to an elective surgical procedure [6]. Our previous findings reported that median values for observed species was 1260 and Shannon diversity was 9.8 in horses admitted for an elective surgery having no gastrointestinal problems and representing a healthy environment. These values were reduced to less than 1000 for observed species and below 9 for Shannon diversity in horses presented for colic [6]. In the current study, the median value for observed species at time 0 when the mares were brought in to a stall from the field was 1500. The number of observed species was unchanged for fed horses throughout the study duration and was reduced to 1200 in horses that were withheld from feed during the late withheld, early refed, and late refed time periods. Shannon diversity in fed horses was between 9.5 and 9.8 which was similar to horses presenting for an elective surgical procedure in our previous study [6]. When feed was withheld from horses, median Shannon diversity was reduced to as low as 8.7 during the early refed time period (Fig. 1). These changes were similar to horses admitted for colic; however, the median duration of colic in that study was only 7 h [6]. Interestingly, horses presented for chronic colic (> 60 h) had species richness of less than 1000 and Shannon diversity of approximately 8.2 which was significantly lower than horses with acute colic [8]. There was less between-horse variation (interquartile range) in the values for richness and diversity for fed horses and horses that were admitted for elective procedure [6] indicating a healthy and consistent composition of gut microbiota. These results were also confirmed by beta diversity analysis visualized on PCoA where shifts in faecal bacterial communities were noted in horses withheld from feed at late withheld and early refed time periods and started to show signs of restoration during the late refed time period (Figs. 2 and 3). Collectively, withholding feed from horses induced change in the faecal bacterial microbiota as early as 10 h post withholding feed. Indications of recovery were observed as early as 12 h post reintroduction of feed. These findings indicate that access to feed maybe a contributing factor to changes in microbiota which may be confounded in gastrointestinal related disorders such as colic. That being said, alterations in the microbiota composition and relative abundance of specific phyla and genera may be more relevant to gastrointestinal disease.

The effect of withholding feed or fasting for prolonged periods was demonstrated in five vertebrate hosts (fish, toads, geckos, quails and mice) showing microbiota changed across gut regions and differences in hosts in response to fasting [22]. The effects of intermittent fasting in humans was reported but no consistent changes were reported [23]. The effects of fasting on microbial changes in the equine gut were not reported. In ruminants, the effect of withholding feed for 24 h was evaluated on changes in rumen microbiota [24]. Interestingly, these authors reported that different species of Prevotella and Anaerovibrio were significantly reduced whereas no changes were reported in cell wall-digesting bacteria such as *Ruminococcus* species and *Fibrobacter* species. Similar findings such as reduction in *Prevotellaceae* and *Anaerovibrio* and no significant changes in fiber-digesting bacteria were observed in this study. In agreement with several reports [24–26], changes in gut microbiota is closely linked to colonic retention time which is increased during fasting leading to increases in *Christensenella* and decreases in rapidly fermenting bacteria due to nutritional deficits.

At the individual bacterial taxa level, in our previous study [6], we identified five bacterial genera that significantly increased and nine genera that decreased in horses that were presented for colic compared to horses admitted to the hospital for an elective procedure. While these results were interesting, we concluded that several other factors including withholding feed may contribute to the dysbiotic condition in the faecal bacterial microbiota of horses with colic. *BS11* and unclassified *Christensenellaceae* were significantly increased in horses presenting for colic [6] and horses withheld from feed in the current study indicating that an increase in these specific bacteria may be attributed to withholding feed and not colic alone. In contrast, increases in *Streptococcus* and *Sphaerochaeta* were unique to horses presented for colic [6, 8] and were not observed in this study indicating that colic is associated with changes in specific genera. Such findings are important to design experiments to fully understand the role of these specific bacterial populations in colic and may suggest markers for developing therapeutic interventions. It has been shown that lineages of unclassified *Christensenellaceae* and unclassified *Dehalobacteriaceae* belonging to Firmicutes are heritable and are also enriched in humans with a lean-body mass index (BMI) phenotype [27], further indicating that these bacteria occupy a unique niche and may contribute to promoting gastrointestinal health. Further studies are needed to fully understand the role of these bacteria and whether their modification can be used to prevent colic or manage recurrent colic.

In contrast, genera such as *Prevotella*, unclassified *Prevotellaceae*, and *YRC22* from Bacteroidetes and *Clostridia* and unclassified *Lachnospiraceae* from Firmicutes, which are considered commensal gut microbiota, were reduced in horses with colic compared to horses admitted for an elective surgical procedure [6]. Importantly, such changes were not observed in the current study; however, we found that unclassified *Marinilabiaceae* and unclassified *Prevotellaceae* from Bacteroidetes, unclassified *Veillonellaceae*, *Anaerovibrio* and *Bulleidia* from Firmicutes, and *GMD14H09* representing Proteobacteria were significantly lower in late withheld, early refed, and late refed time points in horses withheld from feed whereas there were no alterations in these genera in horses that were fed (Fig. 5). Notably, these genera represent rapidly fermenting bacteria that are positively correlated with propionate and succinate production in foregut fermenters [28]. As feed was restricted from horses for a relatively short period of time in this study, changes in faecal microbiota, particularly rapidly fermenting bacteria which rapidly increase with availability of hemicelluloses, were reduced whereas bacterial populations such as unclassified *Clostridiales*, unclassified *Lachnospiraceae*, and unclassified *Ruminococcaceae* were not affected with feed withdrawal for short periods (Supplemental Fig. S3). These three lineages are abundant in the gut microbiome and are important for digestion of plant polymers and maintenance of gut health [29]. It is interesting to note that the commensal microbiota was not affected in horses withheld from feed for 24 h as observed in this study; however, these populations were reduced in horses presented for colic [6]. It can be inferred that while some changes in microbiota at the community as well as individual taxa level noted in horses presented for colic were confounded by withholding feed from horses, there are certain changes in the individual taxa, particularly increases in *Spirochaetes* and *Streptococcus* and decreases in commensal microbiota such as *Prevotella*, unclassified *Prevotellaceae*, unclassified *Clostridia*, and unclassified *Lachnospiraceae* in horses presented for colic that warrant further investigation.

Study limitations include a relatively low number of horses and no sex diversity. Geographic variability was lacking as all horses were housed and sampled in Pennsylvania during the winter season. However, we hypothesize that the overall findings from this study can be extrapolated to other regions and seasons. It is acknowledged that several variables including age, breed, and diet affect the microbiota [30–32]. The mares in this study were mature to geriatric, light horse breeds, fed the same diet, and similarly managed. Despite this, there was clear inter individual variability between mares (Figs. 2 and 3). In previous studies, we identified that each horse had a unique fecal microbial profile and inter-individual variation was greater than any intra-individual variation observed using different sampling collection techniques [33]. In an attempt to address individual variation, each mare was used as their own control.

## Conclusion

Withholding feed has a significant effect on faecal bacterial microbiota diversity and composition particularly following at least 10 h of withholding feed. Diversity and composition began to return toward normal within 24 h of re-introducing feed. The effect of withholding feed should be taken into consideration when interpreting data on the equine faecal bacterial microbiota in horses.

## Methods

### Horses

**Fig. 6:**
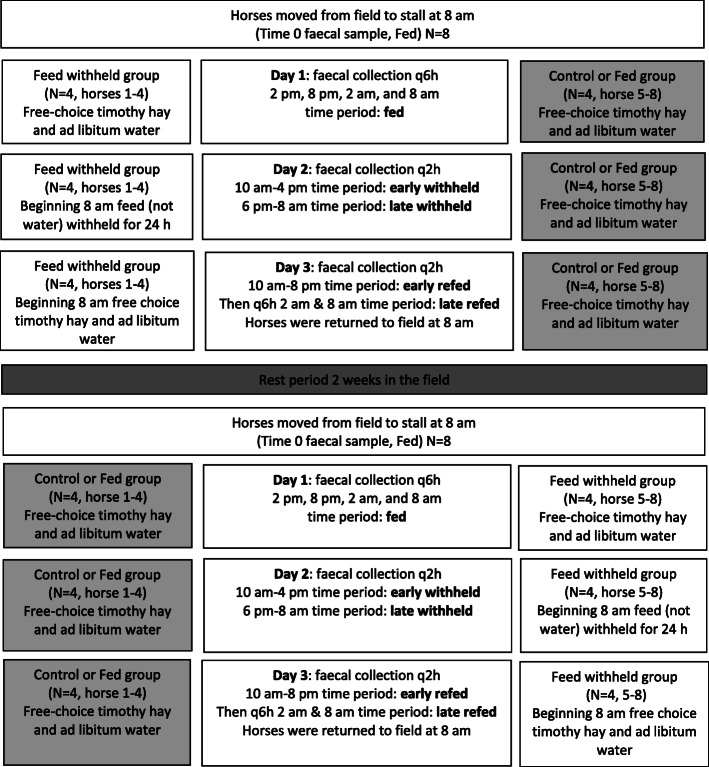
Schematic illustration of the crossover study design

All procedures were approved by the University of Pennsylvania Institutional Animal Care and Use Committee (protocol #806644). A total of 8 Hofmann Center/New Bolton Center-owned mares were used for the study. At the completion of the study the mares were returned to their herd. Because the mares were university-owned, owner informed consent was not required. Each mare was considered an experimental unit and was used as its own control in a crossover study design. A priori sample size calculations were not performed. The mares ages ranged from 14 to 23 years old and breeds included 3 Standardbreds, 2 Thoroughbreds, 1 Quarter Horse, 1 Irish Sport Horse, and 1 Morgan. Three of the mares had previously been routinely ovariectomized for purposes not involving this study. A crossover study design with a 2-week rest period was performed at the Hofmann Center (Fig. 6). Mares were randomly assigned to either fed (control, *n* = 4) or feed withheld (n = 4) groups for the first part of the study. Randomization was performed by blindly selecting the mares’ names from a bag. Potential confounders were not controlled because none were clearly identified and the individuals performing the final sequencing analyses were blinded to experimental group assignment.

### Sample collection

Horses (*n* = 8) were brought from the winter pasture into a stall on the morning of day 0 at 8 am (time 0). An admission physical examination (temperature, heart rate, respiratory rate, mucus membranes, capillary refill time, and borborygmi) was performed for each horse. Physical examinations were performed every 12 h for the duration of the study. Physical examination findings were only reported if they were abnormal.

Due to the crossover study design with 2-week rest period, samples were collected during late winter/early spring during two study time periods: late February-early March and mid-March. The ambient temperature during the first study time period ranged from − 4 °C to 6 °C and during the second from 3 °C to 26 °C. Horses were maintained in a field with little pasture, and their diets were supplemented with free-choice timothy hay due to the scarce forage during the winter season. No other grain supplementation was provided.

Horses were moved into standard-sized box stalls and fed free-choice timothy hay for 24 h and allowed free access to water (day 1) for the acclimation period of the study. Beginning at 8 am on day 2, feed, but not water, was withheld for 24 h from the feed withheld group (*n* = 4) while the control or fed group (n = 4) was allowed continued access to free-choice hay. The horses in the feed withheld group were muzzled; the muzzle allowed for the horses to drink freely. All study horses (feed withheld and control or fed groups) were fed approximately 170 g of Equine Senior (Purina® Equine Senior® Horse Feed) every 6 h during the 24-h feed withheld period for humane reasons and study equivalence. At the completion of the 24-h period during which feed was withheld, the horses in the feed withheld group were reintroduced to free-choice timothy hay at 8 am (day 3).

Faecal samples were obtained from all 8 horses at time 0 (pre) and during the initial 24 h of stall confinement on day 1 at 6-h intervals (time 1.1, 1.2, 1.3, 1.4 [fed]). Sample collection was then performed every 2 h during the period in which feed was withheld and until feed was re-introduced at 24 h on day 2 (time 2.1, 2.2, 2.3, 2.4 [early withheld]; 2.5, 2.6, 2.7, 2.8, 2.9, 2.10, 2.11, 2.12 [late withheld]). Collection was continued every 2 h after reintroduction of feed at 8 am for an additional 12 h until 8 pm (time 2.13, 2.14, 3.1, 3.1a, 3.1b, 3.2 [Early Refed]). Horses were kept in a stall for an additional 12 h and samples were collected at 6-h intervals (time 3.3, 3.4 [Late Refed]) until 8 am the next day at the study conclusion (Fig. 6). Horses were returned to pasture at the completion of the study and allowed a 2-week rest period. At the completion of the 2-week rest period, the study was repeated in a crossover design with horses then being in the alternate group i.e. if horses were in the control fed group in the first part of the study they were in the feed withheld group in the second part and vice versa.

Horses were restrained by a handler for faecal sample collection. Powder-free nitrile gloves (Fisherbrand™) were worn and J-Jelly lubricant (methylcellulose by Jorgensen Laboratories Inc.) was placed on the back of the sampler’s hand as to not contaminate the faecal balls upon sample collection. At least one faecal ball (minimum 100 g) was obtained for sample collection from the horse’s rectum at each collection time. If no faeces were present in the rectum, then no sample was collected for that time point (Supplemental Table S1). The horse’s rectum was not completely evacuated at the time of sample collection. The faecal samples used for analysis were obtained from the center of each faecal ball using a spatula (LevGo Smart Spatula™ Disposable polypropylene). All samples were then placed in a 2 mL microcentrifuge tube (Fisherbrand™ Premium polypropylene) and stored at 4 °C for less than 12 h, and then frozen at − 80 °C until processing.

### 16S rRNA gene PCR and sequencing

Frozen faecal samples were thawed at room temperature and genomic DNA was extracted using a commercially available kit (PSP Spin Stool DNA Plus Kit, Invitek, Berlin, Germany). The extraction involved a bead-beating step prior to following the manufacturer’s guidelines. The extracted DNA was quantified as per the method described by Pitta et al. [34]. The extracted DNA was PCR-amplified for the V1-V2 region of the bacterial 16S rRNA gene using the specific primers F27 (5′-AGAGTTTGATCCTGGCTCAG-3′) and R338 (5′-TGCTGCCTCCCGTAGGAGT-3′) as described in Song et al. [35]. Polymerase chain reaction was performed using a commercial kit (Accuprime Taq DNA Polymerase System, Invitrogen, Carlsbad, CA). The thermal cycling conditions were followed as previously described by Wu et al. [36] Negative controls were included for DNA extraction and PCR amplification. Each amplicon library was quantified, combined to a pool and then sequenced using the Illumina MiSeq platform.

### Bioinformatics and statistical analysis

The raw sequencing data was processed through the QIIME2 (2018.4) pipeline [37]. Briefly, paired end sequence data was de-multiplexed and amplicon sequence variants (ASV) were assigned using the DADA2 plugin [38] with settings that included truncation at 3 frame end of the sequence at 230 nucleotides. MAFFT [39] program was used to generate multiple sequence alignment and to filter highly variable positions with default settings. A phylogenetic tree was constructed using FastTree 2 [40] with default settings. A pre-trained Naive Bayes classifier trained on the Greengenes database (v13.8) for the 16S rRNA gene spanning the V1-V2 region [41] was used for taxonomic classification.

Alpha diversity (Observed ASVs and Shannon diversity) and beta diversity (weighted and unweighted UniFrac distances) were computed using ‘qiime diversity’ plugin. The measured alpha diversity indices were statistically compared between treatment groups or between time periods using the Wilcoxon/Kruskal-Wallis Rank Sum test. A nonparametric permutational multivariate ANOVA (PERMANOVA) test [42] implemented in the vegan package for R was used for beta diversity matrices. Independent variables using in the model were experimental group (fed control v. feed withheld), time period, and the interaction between experimental group and time period. The individual animal identification was included as the random effect variable. Pairwise comparisons were made between groups at each time period.

Analysis of composition of microbiomes (ANCOM) test built in QIIME 2was used to determine differences in bacterial genera between treatment groups. The taxa that showed global significant differences between the two treatments from ANCOM test were further tested for significance at each sampling time point using Wilcoxon rank sum test. A *P* value of 0.05 was used to define significance.

## Supplementary Information

**Additional file 1: Figure S1.** Principle coordinate analysis (PCoA) showing the beta diversity for the effect of horse (Animal ID) and treatment (fed control horses versus feed withheld, FW). (A) weighted (commonly present bacterial populations) and (B) unweighted (presence and absence information of bacteria). Each horse clearly has a distinct microbiota despite having a similar signalment, identical diet, and living in the same environment. There is a significant effect of withholding feed (FW v. control group). The control samples (circles) are more tightly clustered than the FW samples (triangles) and the two groups represent distinct bacterial populations (i.e. FW samples are grouped separately from the control samples). FW, feed withheld.

**Additional file 2: Figure S2**. Principle coordinate analysis (PCoA) showing the beta diversity for horses were outside at pasture compared to inside in a stall. Only fed control horses were used in this analysis and there was no significant effect of moving a horse from the outside pasture to inside a stall. Of note, however, is that horses were fed the same hay outside at pasture and while inside in a stall. (A) Weighted PCoA comparing samples from the time points 0, 1.1, 1.2 (8 am to 8 pm, blue dots) representing the first 12 h from when horses were first moved from outside pasture (outside-pasture) to all other sample time points taken when the horse was inside the stall from 1.3 to 3.4 (day 1 at 2 am to day 3 at 8 am, red dots). (B) Weighted PCoA similar to B except comparing sample collected at time point 0 (ST0, red dots) to all other time points (STothers, blue dots).

**Additional file 3: Supplemental Figure S3**. Relative abundance of unclassified Clostridiales, unclassified Lachnospiraceae, and unclassified Ruminococcaceae at the different time periods (see Fig. 6 for definitions). On ANCOM analysis, there was no significant difference between fed control (black lines) and feed withheld groups (FW, grey lines) and further analysis of individual time points for these genera were not performed.

**Additional file 4: Table S1**. Eight samples that were not collected because of a lack of faeces in the rectum (2% of total samples).

**Additional file 5: Table S2**. Relative abundance of phyla (mean) that were detected at various time points (fed, early withheld [EW], late withheld [LW], early refed [ER], and late refed [LR]) in both horses that received full access to feed (control) and horses that had feed withheld [WH].

**Additional file 6: Table S3**. Relative abundance of genera (mean) that were detected at various time points (fed, early withheld [EW], late withheld [LW], early refed [ER], and late refed [LR]) in both horses that received full access to feed (control) and horses that had feed withheld [WH].

## Data Availability

The bacterial raw sequences have been deposited in the NCBI Sequence Read Archive (SRA) database under BioProject accession number PRJNA645392.

## Abbreviations

DNA: Deoxyribonucleic acid
PCR: Polymerase chain reaction
h: Hour
ASV: Amplicon sequence variants
ANCOM: Analysis of composition of microbiomes
PCoA: Principle coordinate analysis
