# Supplementary figures and images for: Experimental crossover study on the effects of withholding feed for 24 h on the equine faecal bacterial microbiota in healthy mares
Source: BMC Vet Res. 2021 Jan 5;17:3. doi: 10.1186/s12917-020-02706-8 (PMC7786913; doi:10.1186/s12917-020-02706-8)

A) Weighted

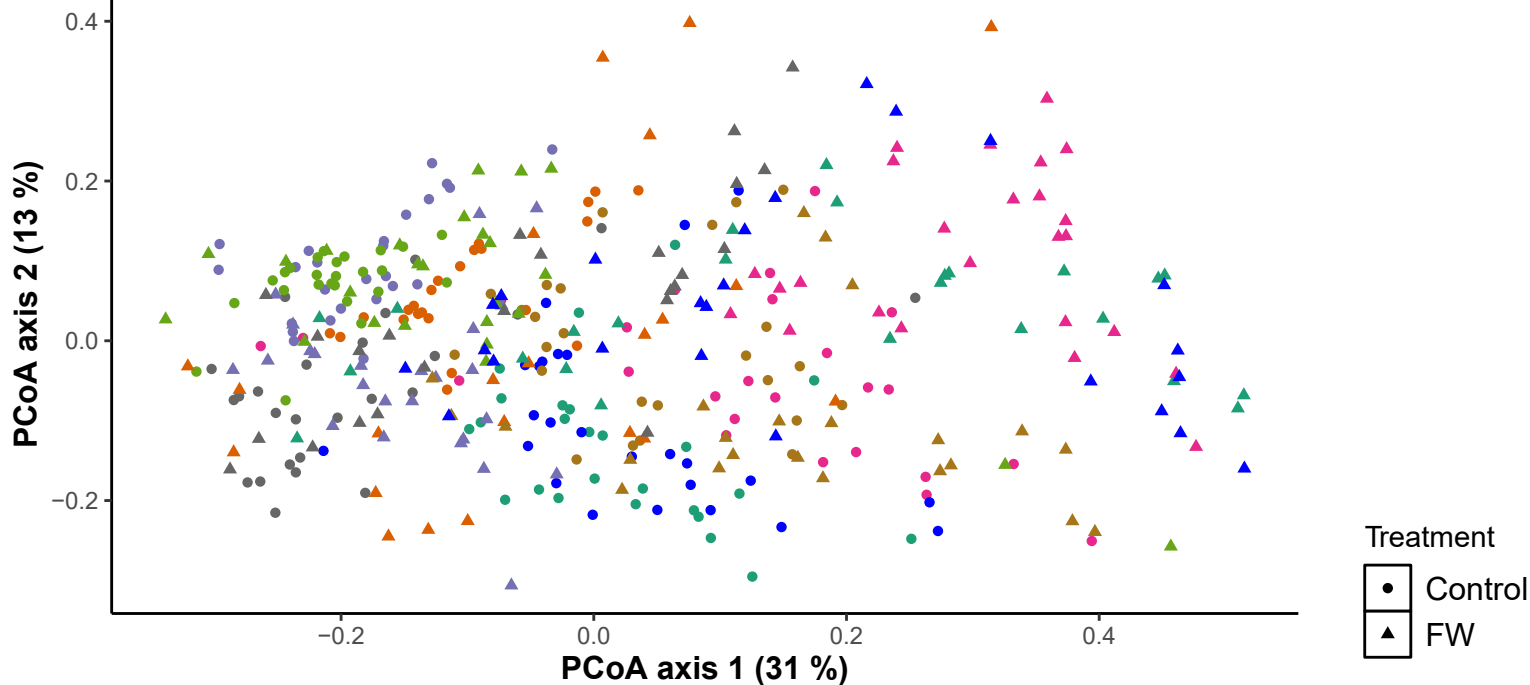

B) Unweighted

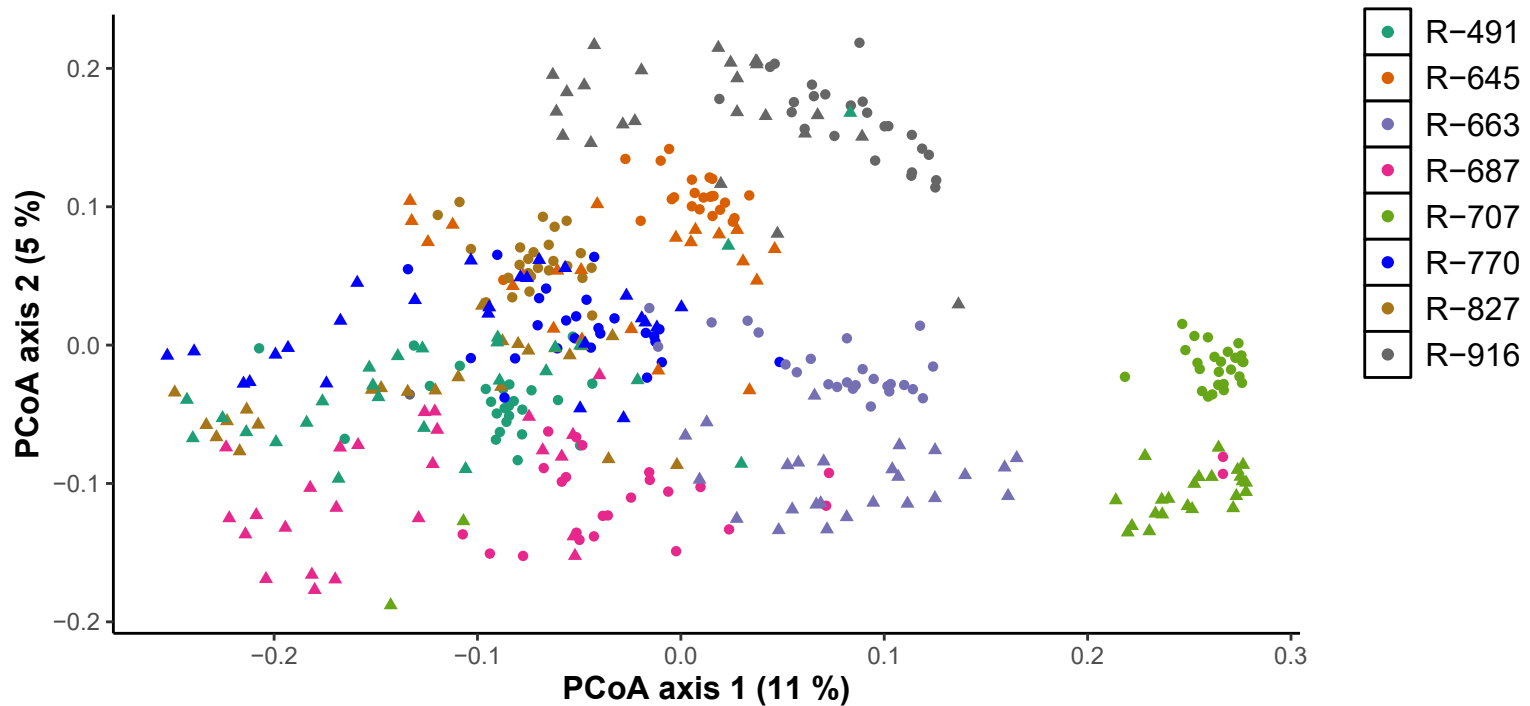

Supplement: Supplementary file 1 — Additional file 1: Figure S1. Principle coordinate analysis (PCoA) showing the beta diversity for the effect of horse (Animal ID) and treatment (fed control horses versus feed withheld, FW). (A) weighted (commonly present bacterial populations) and (B) unweighted (presence and absence information of bacteria). Each horse clearly has a distinct microbiota despite having a similar signalment, identical diet, and living in the same environment. There is a significant effect of withholding feed (FW v. control group). The control samples (circles) are more tightly clustered than the FW samples (triangles) and the two groups represent distinct bacterial populations (i.e. FW samples are grouped separately from the control samples). FW, feed withheld. [file 12917_2020_2706_MOESM1_ESM.pdf]

Weighted-controlOnly

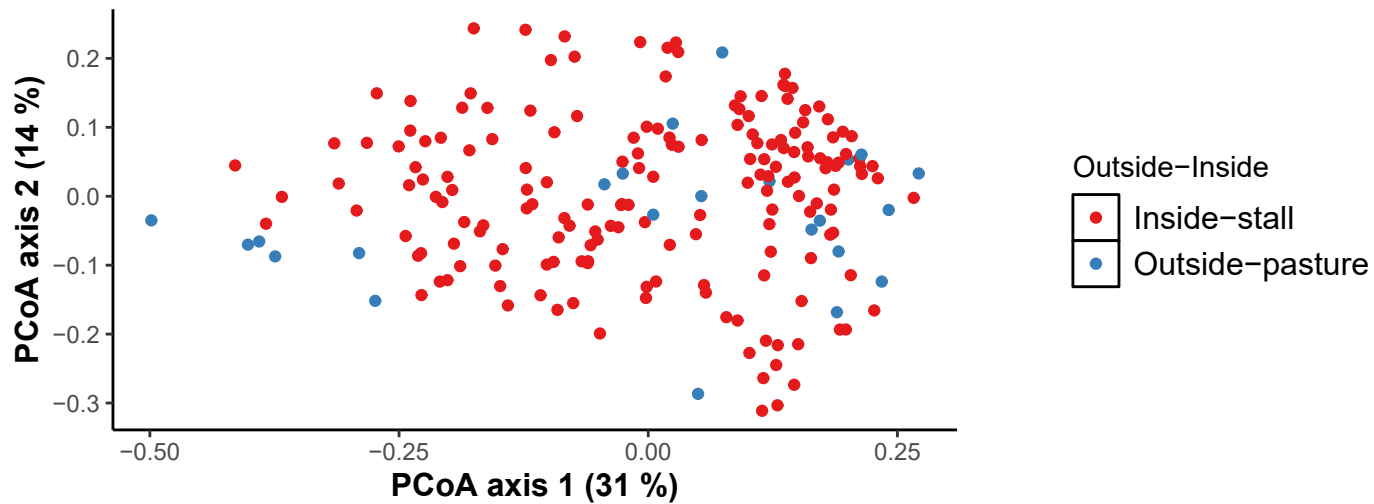

Weighted

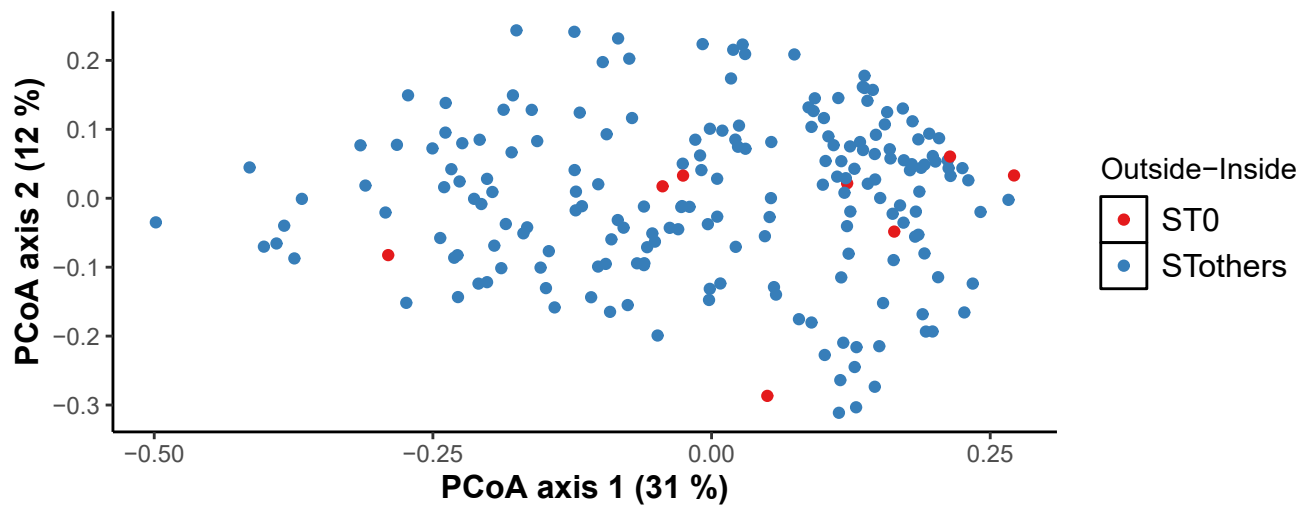

Supplement: Supplementary file 2 — Additional file 2: Figure S2. Principle coordinate analysis (PCoA) showing the beta diversity for horses were outside at pasture compared to inside in a stall. Only fed control horses were used in this analysis and there was no significant effect of moving a horse from the outside pasture to inside a stall. Of note, however, is that horses were fed the same hay outside at pasture and while inside in a stall. (A) Weighted PCoA comparing samples from the time points 0, 1.1, 1.2 (8 am to 8 pm, blue dots) representing the first 12 h from when horses were first moved from outside pasture (outside-pasture) to all other sample time points taken when the horse was inside the stall from 1.3 to 3.4 (day 1 at 2 am to day 3 at 8 am, red dots). (B) Weighted PCoA similar to B except comparing sample collected at time point 0 (ST0, red dots) to all other time points (STothers, blue dots). [file 12917_2020_2706_MOESM2_ESM.pdf]

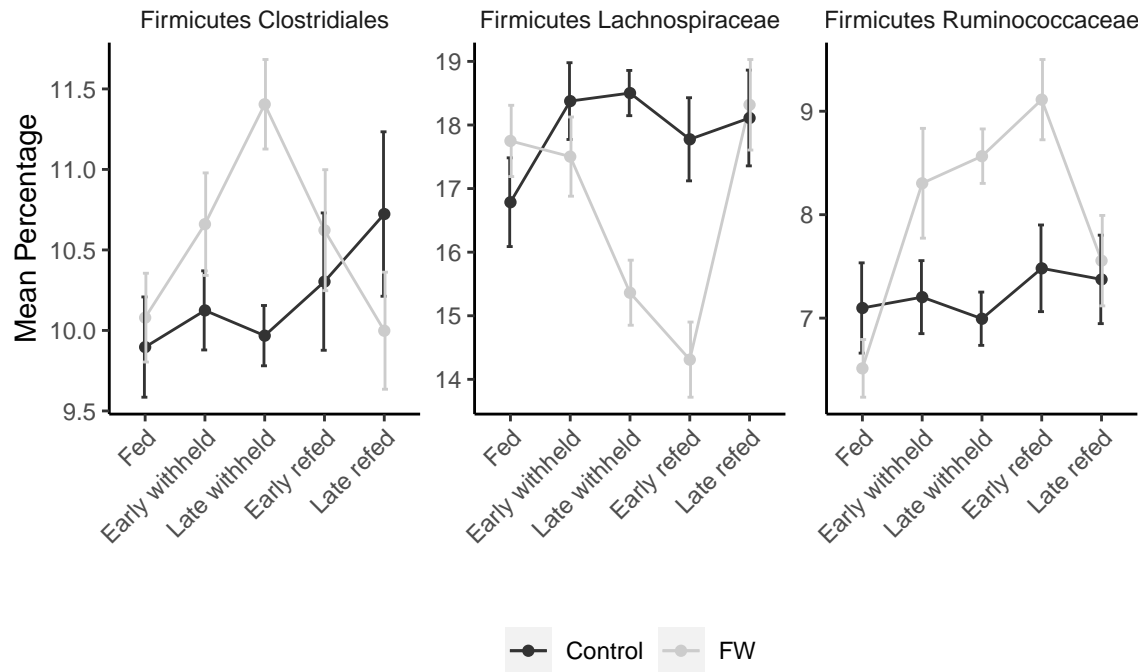

Supplement: Supplementary file 3 — Additional file 3: Supplemental Figure S3. Relative abundance of unclassified Clostridiales, unclassified Lachnospiraceae, and unclassified Ruminococcaceae at the different time periods (see Fig. 6 for definitions). On ANCOM analysis, there was no significant difference between fed control (black lines) and feed withheld groups (FW, grey lines) and further analysis of individual time points for these genera were not performed. [file 12917_2020_2706_MOESM3_ESM.pdf]
