# Supplementary material for: Experimental crossover study on the effects of withholding feed for 24 h on the equine faecal bacterial microbiota in healthy mares
Source: BMC Vet Res. 2021 Jan 5;17:3. doi: 10.1186/s12917-020-02706-8 (PMC7786913; doi:10.1186/s12917-020-02706-8)
Supplement: Supplementary file 4 — Additional file 4: Table S1. Eight samples that were not collected because of a lack of faeces in the rectum (2% of total samples). [file 12917_2020_2706_MOESM4_ESM.docx]

Supplemental Table S1: Eight samples that were not collected because of a lack of faeces in the rectum (2% of total samples).

| Study Period | Experimental Group | Horse | Time Point | Time Period |
| --- | --- | --- | --- | --- |
| 1 | FW | R-645 | 2.6 | Late Withheld |
| 1 | FW | R-645 | 2.7 | Late Withheld |
| 1 | FW | R-645 | 2.13 | Early Refed |
| 1 | FW | R-827 | 2.5 | Late Withheld |
| 1 | FW | R-827 | 2.7 | Late Withheld |
| 2 | FW | R-916 | 2.6 | Late Withheld |
| 2 | C | R-645 | 1.2 | Fed |
| 2 | C | R-645 | 1.4 | Fed |

FW, feed withheld, C, fed control
